# A Case of Insanity Cured by Removal of a Fibroid Tumor of the Ovary

**Published:** 1895-07

**Authors:** Emory Lanphear

**Affiliations:** St. Lovis, Mo., Formerly Professor of Operative Surgery in the Kansas City Medical College, and Professor of the Principles and Practice of Surgery in the St. Louis College of Physicians and Surgeons; 2312 Salisbury St.


### A CASE OF INSANITY CURED BY REMOVAL OF A FIBROID TUMOR OF THE OVARY.

By EMORY LANPHEAR, M. D., PH. D, ST. LOUIS, MO.,

Formerly Professor of Operative Surgery in the Kansas City Medical College, and Professor of the Principles and Practice of Surgery in the St. Louis Co lege of Physicians and Surgeons.

In a recent number of the *Journal of the American Medical Association* I made the assertion that every large insane asylum of the world contains scores of patients who might be cured, and many more who might be greatly improved, by careful examination and proper surgical treatment. This statement was not a pure assumption. I know, first, that the average patient in an insane asylum is examined fairly well at the time of admission, but rarely afterward unless gross evidence of physical disease appears; and only too often in the not remote past no physical examination at all was ever made; and I know, second, that I have in a number of instances operated upon patients who were insane and at the same time affected by some surgical disease like pelvic inflammation, rectal trouble, etc., of an irritative character—and restoration of mind followed convalescence from the operation. A notable example is the following:

Mrs. Nettie B., thirty-six years of age, patient of Dr. T. Brown, of Hamilton, Mo., married at twenty, mother of four children, of which the youngest is six years of age, had for several years a slowly-growing, small, very hard tumor in the pelvis, which pressed upon the bladder, causing marked vesical irritation. For more than a year this tumor seemed to generate serious disturbance at the menstrual periods, and finally typical menstrual mania developed about January, 1891. The maniacal attacks became so severe and so long-continued that she was at last practically a dangerous lunatic all of the time, and was finally so violent that she had to be sent to the hospital for the insane. Whether or not an examination of the pelvic organs was made at the time of her admission I am unable to say, but no treatment was instituted save the usual routine prescription for a tonic and a quieting mixture, with proper feeding, etc. But her family physician was not satisfied—he firmly believed the mental aberration to be dependent upon the pelvic lesion and so, after much trouble and opposition, he had her placed in All Saints' Hospital, in Kansas City, for operation. I first saw her with him November 23, 1891, diagnosticated fibroid tumor of the ovary and had her prepared for cœliotomy.

The operation on November 24, was performed without trouble, the tumor—a true fibroid of the ovary, a rare specimen measuring two and a half by eight inches—being removed with no difficulty, through a three-inch incision. The left ovary was also removed, with its tube, to check menstruation. She was under the anæsthetic a little less than twenty-five minutes. By reason of little chloroform and no hæmorrhage, shock was wholly absent. Convalescence was speedy and uneventful. She sat up in bed on the tenth day, in a chair on the twelfth and returned home on the seventeenth day.

The maniacal excitement did not return until the date for her next menstrual period, though her mind was not exactly right at any time; but she was quite rational most of the time and very docile always; nor was immediate restoration to be anticipated, as was fully explained to her friends. As the month expired she became much excited and at last lost all control of herself for a few days during the period when she would have been menstruating, had the tubes not been removed. This maniacal attack lasted but four or five days and left her in a very satisfactory state. This quiescent condition continued until the next menstrual date when there was once more the same return of the excitement, but to a far less degree than on the previous month. These exacerbations grew less and less as the months passed, and inside of eight or nine months she was declared well and discharged from the asylum.

This improvement was not temporary, as predicted by some physicians. More than three years have elapsed since her dismissal from the asylum, and there has been no indication of a return of the insanity. So I reckon her among my cases of "cures" of mental diseases by surgical treatment. If this case stood alone, I might regard it as an instance of "*post hoc, ergo propter hoc*," but this is only a single report selected from quite a large number of similar character. Enthusiastic as we may become over the influence of bacteria and of toxines in the production of a disease, we cannot and must not ignore the question of reflex irritation as an element in the causation of nervous and mental disorders.

The lesson contained in such a case-history is the necessity for more thorough examinations to ascertain the existence or absence of sources of peripheral irritation; and the advisability of early correction of ovarian and uterine diseases, lacerations, preputial adhesions, nasal obstructions, irritative diseases of the rectum, etc.—in other words the adoption of some of the rules of the "orificial surgeons." These zealous practitioners, though carried to extremes in their enthusiasm, are doing good in directing attention to a class of pathological conditions too long neglected by "regular" doctors; it is to be hoped this influence upon the whole profession may be for good; and not transient.

*2312 Salisbury St.*

